# Homeostasis of Glucose and Lipid in Non-Alcoholic Fatty Liver Disease

**DOI:** 10.3390/ijms20020298

**Published:** 2019-01-13

**Authors:** Hsu-Wen Chao, Shi-Wei Chao, Heng Lin, Hui-Chen Ku, Ching-Feng Cheng

**Affiliations:** 1 Department of Physiology, School of Medicine, College of Medicine, Taipei Medical University, Taipei 11031, Taiwan; chaohw3619@tmu.edu.tw (H.-W.C.); linheng@tmu.edu.tw (H.L.); 2 Graduate Institute of Medical Sciences, College of Medicine, Taipei Medical University, Taipei 11031, Taiwan; 3 Ph.D. Program in Biotechnology Research and Development, Taipei Medical University, Taipei 11031, Taiwan; south102411@gmail.com; 4 Department of Pediatrics, Taipei Tzu Chi Hospital, Buddhist Tzu Chi Medical Foundation, New Taipei City 23142, Taiwan; 5 Department of Pediatrics, Tzu Chi University, Hualien 97004, Taiwan

**Keywords:** non-alcoholic fatty liver disease, glucose, lipid

## Abstract

Industrialized society-caused dysregular human behaviors and activities such as overworking, excessive dietary intake, and sleep deprivation lead to perturbations in the metabolism and the development of metabolic syndrome. Non-alcoholic fatty liver disease (NAFLD), the most common chronic liver disease worldwide, affects around 30% and 25% of people in Western and Asian countries, respectively, which leads to numerous medical costs annually. Insulin resistance is the major hallmark of NAFLD and is crucial in the pathogenesis and for the progression from NAFLD to non-alcoholic steatohepatitis (NASH). Excessive dietary intake of saturated fats and carbohydrate-enriched foods contributes to both insulin resistance and NAFLD. Once NAFLD is established, insulin resistance can promote the progression to the more severe state of liver endangerment like NASH. Here, we review current and potential studies for understanding the complexity between insulin-regulated glycolytic and lipogenic homeostasis and the underlying causes of NAFLD. We discuss how disruption of the insulin signal is associated with various metabolic disorders of glucoses and lipids that constitute both the metabolic syndrome and NAFLD.

## 1. Introduction

Non-alcoholic fatty liver disease (NAFLD) has become the most common chronic liver disease worldwide because of its complex pathogenesis and difficulty in diagnosis. Epidemiological studies indicate that the prevalence of NAFLD is increasing worldwide with each passing year, with the incidences of NAFLD around 30% and 25% of people in Western and Asia countries, respectively [1,2]. In the United States and Europe (particularly in the countries of Germany, France, Italy, and the United Kingdom), between ~52 and 64 million people are currently have NAFLD, with annual direct medical costs of about $103 billion and €35 billion, in the United State and Europe, respectively [3]. Non-alcoholic fatty liver disease includes two types of conditions: non-alcoholic fatty liver (NAFL) and non-alcoholic steatohepatitis (NASH) [4,5]. Non-alcoholic fatty liver is defined by greater than 5% of steatosis of parenchyma in the liver, in the absence of any significant inflammation or fibrosis (scarring). However, NASH usually displays lobular inflammation and hepatocyte ballooning histologically with injured hepatocyte in a background of steatosis, which is associated with faster fibrosis progression than NAFLD [5,6,7]. Furthermore, particular stains such as cytoplasmic keratin (KRT) 8/18 immunohistochemistry may detect ballooned hepatocytes. A recent study by Golob-Schwarzl et al. [8] found that a high KRT8/18 ratio caused the change in hepatic lipid profile resulting in ballooning with a Mallory–Denk body (MDB) formation. Numerous risk factors, such as metabolic derangement, obesity, and insulin resistance (IR) lead to liver accumulation of triglycerides and free fatty acids and growing of NAFLD [9]. Overeating, consumption of energy dense, nutritionally imbalanced and appetite dysregulating food have been reported to be associated with NAFLD, and lifestyle modification usually can improve hepatic steatosis and liver fibrosis [10]. In addition, NAFLD have so far been linked with inherited factors, such as polymorphisms of genes for lipid transport and lipid metabolism [11]. In this review, we summarized the current and potential mechanisms for generation of NAFLD. We will provide context for how disruption of the insulin signal is associated with various metabolic disorders of glucose and lipid that constitute the metabolic syndrome and NAFLD.

## 2. Two-Hit and Multiple-Hit Hypotheses

The underlying mechanism for the development and progression of NAFLD is complex and multi-factorial. In 1998, Day and James [12,13] first proposed that NAFLD may be considered as a disease with a “two-hit” process of pathogenesis with lipid peroxidation-mediated liver injury. In the patients with a sedentary lifestyle, obesity or IR, an increased influx of free fatty acid (FFA) to the hepatocyte was observed in the liver [14]. By esterification, FFA and glycerol can be further transformed into triglycerides (TGs) and stored in hepatocytes. The first hit for NAFLD development was the generation of hepatic steatosis through aberrant accumulation of TGs in hepatocytes, called steatosis which is the hallmark feature in NAFL. Accumulation of TGs in hepatocytes abnormally increases the risk of the liver to various chances for “second hits”, which in turn results in cellular death, inflammation, and fibrosis characteristics of NASH. For the second hit, it can be a variety of factors, such as elevation of oxidative or endoplasmic reticulum (ER) stress, proinflammatory cytokines, mitochondrial dysfunction, and gut-derived bacterial endotoxin, which in turn leads to steatohepatitis and/or fibrosis [13,15]. The “two-hit” hypothesis has now been explained, as it has been believed that many factors may cause the development of NAFLD simultaneously, which supports the “multiple hit” model proposed in 2010 [16]. Then, evidence emerged which has supported that FFA can directly induce toxicity through increasing oxidative stress and by activation of inflammatory pathways [17]. Additionally, inhibiting triglyceride synthesis improves hepatic steatosis, but exacerbates liver damage and fibrosis in obese mice with NASH [18]. In the liver, replacement of dead hepatic cells by stimulating replication of mature hepatocyte can reconstitute the normal function of the liver, which has been reported to be inhibited by oxidative stress and results in expansion of hepatocyte-like cell populations in the liver [19]. Loss of hepatocyte numbers gradually promotes accumulation of hepatocyte-like cells, which has been implicated in chronic liver injury for development of fibrosis, cirrhosis, and hepatocellular carcinogenesis [19]. Indeed, many suggested hit factors can interact with each other and are considered as a malicious circle to induce NAFLD, which provides a more accurate explanation of NAFLD pathogenesis [20]. Recent advances in genome-wide association studies have found several promising candidate genes which play the genetic background for the disease. Moreover, the interplay between diet and gut microbiota can play another critical role in the development of NAFLD. Although many factors, especially a high-fat diet, inactive lifestyle, and genetic variants have been shown to be involved in the progression of NAFLD, IR is considered to play a key role in the formation of NASH, which lead to hepatic de novo lipogenesis, reduction of lipolysis of adipose tissue, and consequent elevation of FFA in the liver [21].

## 3. Glucose Homeostasis and NAFLD

Non-alcoholic fatty liver disease is the hepatic manifestation of metabolic syndrome and defined as the presence of the following symptoms: (1) increase in waist circumference, (2) hypertriglyceridemia, (3) hypertension, (4) fasting hyperglycemia, and (5) low high-density lipoprotein (HDL) level [22]. Insulin resistance is the key pathogenic feature of the metabolic syndrome and is now recognized as the most common risk factor for NAFLD development and progression [23,24,25]. Two types of insulin resistance, systemic and hepatic insulin resistance, are defined as the decreased ability of tissues to respond to insulin signals. Systemic insulin resistance is characterized by the inability of insulin to reduce blood glucose levels appropriately because of the impaired translocation of the GLUT4 receptor to the surface membrane of the muscle cell and leading to loss of insulin-dependent uptake of glucose [26]. Hepatic insulin resistance is caused by interrupting insulin-induced suppression of hepatic glucose production but raising preserved stimulation of lipogenesis conversely [26]. For patients with insulin resistance, the islets of Langerhans are stimulated to promote insulin secretion to overcome the defect in plasma glucose absorption and to reduce glucose generation in liver. As the production rates of glucose are high aberrantly in hepatocyte in the presence of high insulin level, it is characterized as a sign of hepatic insulin resistance [27]. Glucose metabolism is tightly regulated by insulin which is in turn regulated by glucose concentration in plasma after a meal or hormone release [28]. In the muscle and liver, insulin promotes glucose uptake for glucose oxidation or glycogen storage. Insulin also controls lipid metabolism, as it enhances fatty acid re-esterification into triglyceride in adipocytes and the liver. The main function of insulin in the liver is to suppress glycogenolysis and gluconeogenesis. Conversely, insulin promotes hepatic cholesterol and triglyceride synthesis and glucose uptake into muscle and fat. As hepatocytes lose insulin-mediated autoregulation, such as in LIR gene deleted mice, glucose production is increased from both pathways [29]. However, in a type 2 diabetes liver, not only higher levels of glycogenolysis and gluconeogenesis are detected, but also elevation of cholesterol and triglyceride synthesis are observed, suggesting that these pathways still are insulin sensitive [30]. Importantly, the substrate provided from insulin-resistant adipocyte contributes the additional triglyceride synthesis in the liver [31]. This paradox in hepatic insulin resistance is that insulin fails to suppress hepatic glucose production, yet it continues to stimulate lipogenesis, resulting in hyperglycemia, hyperlipidemia, hepatic steatosis, and type 2 diabetes [30,32].

Insulin-mediated activation (phosphorylation) of insulin receptor substrate (IRS) proteins has been recognized as the major basis for linking insulin resistance to NAFLD. The activation of IRS has two main signaling pathways: the phosphatidylinositol 3-kinase (PI3K)-AKT/protein kinase B (PKB) pathway and the Ras-mitogen-activated protein kinase (MAPK) pathway [33]. The MAPK pathways are not involved in regulating metabolic actions of insulin but rather in facilitating mitogenic and growth effects of insulin. Most of the insulin-mediated metabolic actions are controlled by the PI3K–AKT/PKB pathway, which is phosphorylated by the insulin receptor through two major substrates, IRS-1 and IRS-2 [34]. By binding to insulin through two extracellular subunits, the activity of the insulin receptor is turned on by the trans-phosphorylation of its β subunits followed by auto-phosphorylation at other tyrosine residues in the adjacent membrane regions and results in the increased catalytic activity of the kinase [35]. Then, the activated IR phosphorylates tyrosine residues on intracellular substrates, such as IRS1, IRS2, and Shc isoforms, and recruits subtracts to a juxtamembrane region in the receptor containing an NPXY motif. Upon phosphorylation, these substrates interact with a series of effectors with Src homology 2 (SH2) domains including p85 regulatory subunit of PI 3-kinase (PI3K) which generates the lipid products phosphatidylinositol bisphosphate (PIP_2_) and phosphatidylinositol 3,4,5-trisphosphate (PIP_3_) through the catalytic subunit p110 [36]. Binding to PIP_2_ and PIP_3_ is required for the recruitment and activation of phosphoinositide-dependent kinase-1 (PDK1) and its substrate kinases Akt/PKB via the PH domains, which plays an essential role in insulin-stimulated glucose uptake and glucose transporters (Gluts) translocation in liver and adipose tissue [37].

Insulin plays a primary role for decrease glucose level in plasma by controlling hepatic glucose metabolism. Through the Akt/PKB-signaling pathway, insulin promotes glycolysis and glycogenesis for glucose utilization. It has been known that the Akt/PKB-mediated glucose synthase kinase 3 (GSK-3) signal increases glycogenesis by activating glycogen synthase. Insulin also enhances the activity of glycogen synthase by upregulating protein phosphatase 1 (PP1), which is a Ser/Thr protein phosphatase for dephosphorylation and activation of glycogen synthase [38]. In addition, insulin is also a key inhibitor of hepatic glucose production by suppression of glucose production through glycogenolysis (glycogen breakdown) and gluconeogenesis (de novo glucose production) [39]. In the liver, glucose monomers are removed from glycogen as glucose-1-phoaphate by phosphorylated glycogen phosphorylase and are released into the plasma for the utilization by other cells via GLUT2. Under insulin stimulation, glycogenolysis is inhibited by activating PP1 and phosphodiesterase. PP1 dephosphorylates glycogen phosphorylase directly, and phosphodiesterase converts cAMP to AMP, thus inactivating PKA and suppressing the phosphorylation of glycogen phosphorylase. Moreover, insulin also suppresses hepatic gluconeogenesis which is defined as the synthesis and release of glucose from non-carbohydrate precursors, such as lactate, glycerol, and glucogenic amino acids. Insulin redistributes carbons derived from gluconeogenic flux to glucose-6-phosphate (G6P) to hepatic glycogen rather than blood glucose for inhibition glucose level [40,41]. Therefore, insulin resistance induces an elevation of glycogenolysis and an increase in hepatic glycolytic intermediates, which leads to raised glycolysis and an inhibition of gluconeogenic flux to G6P by concurrent hyperglycemia [42]. In addition, evidence from mouse models have supported that a deficiency of genes involved in insulin-mediated signals result in insulin resistance and glucose intolerance [43,44,45,46].

Moreover, genetic variations involved in insulin signaling have been associated with NAFLD pathogenesis and progression [47]. Around 28.7% and 18.1% of people associated with increased body weight/dyslipidemia and diabetes display the ectonucleotide pyrophosphatase/ phosphodiesterase1 (ENPP1) Lys121Gln (rs1044498) and IRS1 Gly972Arg (rs1801278) polymorphisms, respectively [47]. A gain-of-function mutation in ENPP1 Lys121Gln inhibits insulin receptor activity and causes insulin resistance and accelerates liver fibrosis in a cohort of obese NAFLD patients. In contrast, a loss-of-function mutation in IRS1 Gly972Arg results in hyperglycemia and associates with increased hepatic fibrosis. The ENPP1 121Gln SNP was significantly associated with higher prevalence in patient of moderate/severe fibrosis stage, and these patients also had higher prevalence of 972Arg IRS-1 polymorphism as well. Importantly, both polymorphisms have a marked reduction of ~70% of AKT activation status and cause higher risk of insulin resistance in obese patients with NAFLD [47].

## 4. Lipogenesis and NAFLD

Triglycerides are the major form of fat to be accumulated in the liver of patients with NAFLD, which can derive from esterification of glycerol and FFAs for storage or secretory pools [48]. Using the triglyceride-to-high-density lipoprotein cholesterol ratio, high triglycerides has been used to predict the existence of insulin resistance [49]. The accumulation of triglycerides in the liver (hepatic fat deposition) occurs as an abnormal balance between the hepatic de novo lipogenesis, hepatic availability of FFAs (hepatic lipolysis), and lipid export in the form of triglyceride-rich very-low-density lipoprotein (VLDL) from the liver happens. Free fatty acids can be generated from several pathways including increased hepatic fatty acid synthesis (de novo lipogenesis), delivery of FFAs from adipose tissue lipolysis, delivery of dietary fat, and reduced FA oxidation [50]. In hepatocyte, FFAs go into acyl-coenzyme A (CoA) synthases activity and become fatty acyl-CoAs, which undergo either esterification or β-oxidation pathways [51]. Actually, in mouse models, accumulation of triglycerides is not hepatotoxic in itself and can be a defective mechanism to balance redundant FFAs [18,52]. However, DGAT2 causes reduction of triglycerides in hepatocyte and eventually enhances FFAs oxidation thus deteriorating steatohepatitis [18]. Hence, elevation of triglyceride levels is considered to happen simultaneously with toxic metabolite formation, lipotoxicity, and liver injury and indicates that the system for turning food into energy is not working properly [53]. It is important that dysregulation of lipolysis and de novo lipogenesis are particularly a critical effect for the significant association between risk of insulin resistance and NAFLD. 

Adipose tissue is the main organ for energy storage, and insulin is the major factor in the inhibition of lipolysis of adipocyte. In plasma, fatty acids are under the form of triglycerides (an ester derived from three fatty acids on glycerol) carried by different lipoproteins such as chylomicrons (derived from the gut) or very low-density lipoproteins (produced in the liver). During energy supplies, vascular endothelium in adipose tissue absorbs and hydrolyzes triglycerides into non-esterified fatty acids by insulin-activated lipoprotein lipase. Insulin also promotes the translocation of the glucose transporter GLUT4 to the plasma membrane of adipocyte for glucose uptake. As released fatty acids are conveyed into adipocyte by fatty acids transporters, fatty acids are then re-esterified using glycerol 3-phosphate derived from glucose as a backbone to form triglycerides and stored within lipid droplets [54]. On the contrary, for energy requirements during fasting or exercise, lipolysis is conducted to hydrolyze triglycerides into fatty acid and glycerol through adipose triglyceride lipase hydrolyzation cascade. Subsequently, released fatty acid could be oxidized in muscles and glycerol is transported into the liver to be the precursor of glyconeogenesis [55].

Glucose from excess dietary carbohydrate undergoes glycolysis in liver and is eventually converted into fatty acids to be esterified to triglycerides for very low-density lipoproteins secretion. In the physiological condition, insulin controls hepatic glucose production through regulating lipolysis of adipose tissues, and thus reducing fatty acid influx to the liver [56]. Then, the availability of hepatic acetyl-CoA concentrations and pyruvate carboxylase activity are reduced and leads to consequently decreased conversion of pyruvate to glucose in the liver. However, in NAFLD patient with insulin resistance, the accelerated lipolysis in adipose tissue causes inappropriately enhanced hepatic glucose production, which further upregulates hepatic de novo lipogenesis. Enhanced de novo lipogenesis abnormally has been reported in patients with insulin resistance and contribute to a critical biochemical pathway for the pathogenesis of NAFLD [57]. Hepatic de novo lipogenesis is a biosynthetic pathway for the generation of fatty acid from acetyl-CoA, which is provided with substrate primarily through glycolysis and the metabolism of carbohydrates [58,59]. Therefore, healthy normo-insulinemic individuals with a high carbohydrate diet or a patient with insulin resistance can promote the de novo lipogenesis because of an excessive substrate loading in the liver [60].

Additionally, genetic variations in the patatin-like phospholipase domain containing 3 (*PNPLA3*) gene has been reported to be associated with high susceptibility to NAFLD [61,62]. A genome-wide study demonstrated that the I148M allele (rs738409: C>G) of the *PNPLA3* gene displays a cytosine to guanine substitution and leads to an isoleucine to methionine switch at codon 148 [61]. A higher hepatic triglyceride level and an elevated serum level of alanine aminotransferase (ALT) were identified in people with homozygous for the G allele. Notably, evidence showed that *PNPLA3* (rs738409: C>G) may also play a role in influencing fibrosis severity in patients with fatty liver. Similarly, the variant rs58542926 E167K in transmembrane 6 superfamily member 2 (*TM6SF2*) gene which is involved in VLDL secretion, has been associated with the pathogenesis of NAFLD and with liver disease severity [63]. Most recently, the polymorphism rs641738 in membrane bound O-acyltransferase domain containing the 7 (*MBOAT7*) gene was identified as the new risk factor for NAFLD, and was also associated with severity of fibrosis in alcoholic liver disease [64]. These studies indicate that *PNPLA3*, *TM6SF2*, and *MBOAT7* variants might be highly associated with liver injury in patients with NAFLD. 

## 5. Insulin-Induced Regulator in NAFLD

The transcriptional effects of insulin are mediated by a complex insulin-signaling network and correlate to multiple biological phenomena. In the liver, the insulin-induced PI3K/Akt pathway activates the transcription factors which control the transcription of the genes encoding metabolic enzymes of hepatocyte. In response to insulin stimulation, lipogenic genes can be activated through transcriptional level after a meal. Over hundred genes at the transcriptional level can be controlled by insulin-mediated signals [65,66]. The four major transcription factors involved insulin-controlled lipogenesis are upstream stimulatory factors (USFs), sterol regulatory element binding protein-1c (SREBP-1c), carbohydrate responsive element binding protein (ChREBP), and Liver X receptor alpha (LXRa). Both USF-1 and -2 heterodimer have been reported to be required for fatty acid synthase (FAS) promoter activation under insulin controlling, which is a central enzyme of lipogenesis [67]. Upstream stimulatory factors belong to basic helix–loop–helix leucine zipper (bHLH-LZ) transcription factors for transcriptional activation through binding an E-box (5′-CANNTG-3′) at target gene promoter. The essential function of USFs in lipogenesis has been proofed in USF-1 or USF-2 knockout mice which display impaired activation of lipogenic gene in vivo upon high carbohydrate diets [68]. In humans, single nucleotide polymorphism (SNP) analysis has suggested USF-1 as a critical candidate of familial combined hyperlipidemia (FCHL) [69]. In this regard, mouse models with overexpression of USF in the liver may help to investigate the role of USF in hepatosteatosis and insulin resistance. 

However, USF binds to the FAS promoter in both fasted and fed states constitutively [70]; it has been revealed that USFs inhibit or activate the FAS promoter by recruiting various cofactors during fasted and fed stats. The SREBP-1c, another basic-helix–loop–helix leucine zipper (bHLH-LZ) transcription factor, was originally identified as a transcription factor binding to the sterol-regulatory element (SRE) for cholesterol and lipid regulation, which cooperate with USFs for activation of FAS promoter transcription [70,71]. The SREBP family genes are considered to regulate genes involved in controlling cholesterol homeostasis and de novo fatty acid synthesis [72]. Although some functional overlap among the three SREBP variants have been demonstrated, these proteins control different metabolic pathways [73]. The SREBP-1a transactivates genes involved in both lipogenic and cholesterogenic, whereas SREBP-2 regulates genes for cholesterol biosynthesis and metabolism. Sterol regulatory element binding protein-1c preferentially influences the transcription of genes that regulate de novo lipogenesis specifically in liver and adipose tissue [74]. Importantly, elevation of nuclear SREBP-1c level in hepatocyte has been indicated to mediate the development of hyperlipidemia in type 2 diabetes and hyperinsulinemia [75]. The activation of SREBP-1c is promoted by insulin-mediated PI3K/PKB signaling, which causes phosphorylation and proteolytic release of the membrane-bound immature form SREBP-1c from the Golgi membrane to become the active form stat [76,77]. The inhibition of PKB and PI3K has demonstrated that reduced phosphorylation and processing of SREBP-1c [76]. Moreover, constitutively active PKB in hepatocytes causes upregulation of mammalian target of rapamycin complex 1 (mTORC1) signal, another critical interconnection of PI3K/PKB pathway, and leads to elevation of the mature form of SREBP1c and rates of de novo lipogenesis [78]. Moreover, the insulin-induced proteolytic activation of SREBP-1c needs the involvement of two ER membrane protein SREBP cleavage-activated protein (SCAP) and NR1D1-mediated circadian signaling via insulin induced gene (INSIG) [79,80]. Insulin enhances the affinity of SCAP/SREBP-1c complex for coatomer protein complex II (COPII) vesicles and rapidly proteolytic processing of the nascent SREBP-1c, which is processed via a two-step cleavage by site 1-protease (S1P) and site 2-protease (S2P) within the Golgi apparatus [30,79]. In addition, the sterol-sensing domain of SCAP also interacts with a regulator of cholesterol biosynthesis, INSIG1, to make the SCAP/SREBP complex stay longer in the ER, which prohibits SCAP from carrying activated SREBP to the Golgi complex and ultimately blocks SREBP from acting as a transcription factor [81]. The active SREBP-1c then translocates into the nucleus where it promotes transcription of lipogenic genes through binding to the SRE on the target gene promoter or cooperation with USFs. Deletion of *Srebp-1c* alone can reduce around 50% refeeding-induced upregulation in levels of hepatic lipogenic mRNAs and speeds of fatty acid synthesis [82]. Upregulation of *Srebp-2* is a partially compensatory pathway for activation the lipogenic mRNAs in the loss of *Srebp-1c* in liver, which is completely abolished as the hepatic activity of all three SREBPs is blocked [83]. 

However, SREBP-1c alone is not adequate to maintain the synergistic induction of glycolytic and lipogenic genes in response to both insulin and glucose [84,85]. According to promoter-mapping, most lipogenic genes, such as FAS and acetyl-CoA carboxylases (ACCs), not only have SER for SREBP-1c binding, but also contain the carbohydrate responsive element (ChoRE) that interacts with the carbohydrate responsive element binding protein (ChREBP) [86]. These two pathways are induced by elevation of insulin and glucose signal. Interplay between ChREBP and SREBP-1c has been reported to coordinate postprandial glycolysis and lipogenesis in livers [87]. Although ChREBP is a critical glucose-activated transcription factor, it regulates approximately 50% of de novo lipogenesis in the liver [88]. ChREBP is detected in the liver, white adipose tissues (WATs), brown adipose tissues (BATs), the intestine, muscle, and pancreatic β-cells, which has α and β isoforms [89]. ChREBPα distributes mainly in the cytosol, which is translocated into the nucleus upon glucose stimulation, and then induces transcription of ChREBPβ. ChREBPα has a low glucose inhibitory domain (LID) and a glucose response conserved element (GRACE); however, ChREBPβ has only GRACE. Low glucose inhibitory domain suppresses GRACE-mediated ChREBPα activity under low-glucose conditions, which is an important key step to make the glucose threshold for ChREBP-mediated gene expression and makes ChREBP a “glucose sensor”. Whereas, ChREBPβ is constitutively active under any glucose conditions and has more potent transactivity than ChREBPα. ChREBP is also a bHLH-LZ transcription factor, and the major function of ChREBP is regulation of fructose metabolism and regulates the expression of genes involved in monocarbohydrate transport, glycolysis (Glut2, liver pyruvate kinase), fructolysis (Glut5, ketohexokinase), and de novo lipogenesis (acetyl CoA carboxylase, fatty acid synthase) [90,91]. Deletion of *Chrebp* suppresses high sucrose diet-induced and leptin-deficient obesity, because *Chrebp*^−/−^ mice cannot consume fructose or sucrose efficiently [90]. Moreover, hepatic deletion of *Chrebp* reduced the basal mRNA level of genes for glycolytic and lipogenic processes and prevented the induction of these genes in response to sucrose. Recently, a study in liver specific (L)-*Chrebp*^−/−^ mice with restoration of *Srebp-1c* demonstrated that the sucrose-induced activation of a subset of lipogenic genes were recovered, but it had no effect on glycolytic genes [87]. In turn, *Chrebp* failed to normalize the postprandial induction of lipogenic genes in mice without *Srebps* genes expression. These results indicated that coordination of *Srebp-1c* and *Chrebp* are necessary for the induction of glycolytic and lipogenic mRNAs in response to excess carbohydrates. Notably, SREBP-1c involves insulin-induced activation of lipogenic genes, whereas ChREBP mediates glucose-induced both glycolytic and lipogenic genes, which provides a critical mechanism to maintain the lower synthesis of fatty acids until the stimulation of insulin and glucose.

**Figure 1:**
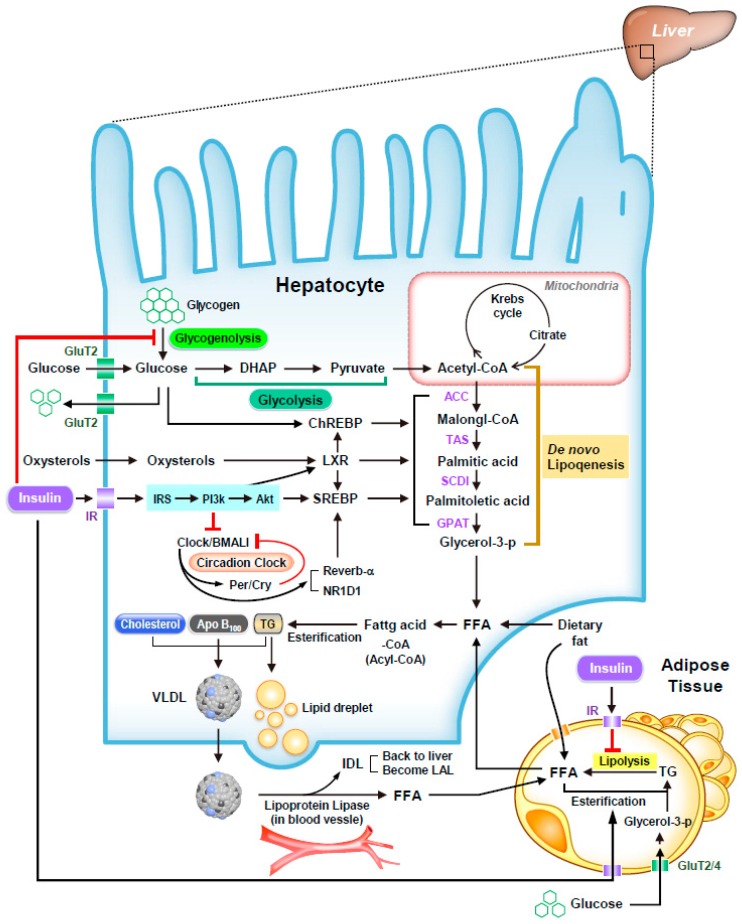
The hepatic metabolism of lipid and carbohydrate through insulin induced transcriptional regulation and circadian clock. Insulin signals and insulin-induced regulators: The schematic depicts the synergistic action of insulin and glucose in regulating lipogenesis in the liver and adipose tissue. Most of the insulin-mediated metabolic actions are controlled by the PI3K-AKT/PKB pathway. Under insulin stimulation, glycogenolysis is inhibited by activating PP1 and phosphodiesterase. Insulin activates the transcription of *Srebp-1c* and the proteolytic processing of SREBP-1c protein. The insulin-induced *Srebp-1c* transcription is facilitated by LXR and SREBPs themselves. LXR promotes lipogenesis not only primarily by increasing *Srebp-1c* expression, but also directly activate the promoters of *Chrebp* and some lipogenic genes. ChREBP is also activated by glucose signal by multiple mechanisms for the further progression of lipogenesis. Metabolism of glucose and lipid: After diet, vascular endothelium in adipose tissue absorbs and hydrolyzes triglycerides into non-esterified fatty acids by insulin-activated lipoprotein lipase. As released fatty acids are conveyed into adipocyte by fatty acids transporters, fatty acids are then re-esterified using glycerol 3-phosphate derived from glucose as a backbone to form triglycerides and stored as lipid droplets. Whereas fasting will promote lipolysis to convert triglycerides within lipid droplets into free fatty acid which is then uptook by hepatocytes for energy production. Insulin also promotes the absorption of glucose transporter to the plasma membrane of adipocyte for glucose uptake. However, insulin resistance causes the accelerated lipolysis in adipose tissue inappropriately and enhances hepatic glucose production through glycogenolysis, which further upregulates hepatic de novo lipogenesis and contributes to a critical biochemical pathway for the pathogenesis of NAFLD. Cross-talk between insulin and circadian clock: Growing evidences show a strong link between circadian clock with energy homeostasis. Hepatic circadian clock is regulated by insulin and altered by insulin resistance. In turn, circadian clock plays an essential role for regulating insulin secretion in the pancreas and balancing blood sugar levels. Insulin-induced regulators, such as SREBP-2, ChREBP, and lipogenic genes, also are directly targeted by circadian clock that drives the rhythmic expression of master regulators and rate-limiting enzymes of key hepatic metabolic outputs.

In addition to SREBP-1c and ChREBP, liver X receptors α and β (LXRα and LXRβ) also play pivotal roles for maintenance the rates of lipogenesis. Upregulation of SREBP-1c and ChREBP has been speculated to be the principal regulators of NAFLD formation, which is induced by insulin resistance and transcriptionally controlled by LXRa [92]. Accumulated evidence has shown that expression of *Srebp-1c* and *Chrebp* is regulated by LXRs which are ligand-activated nuclear receptors with important functions in the transcriptional control of lipid metabolism such as fatty acid elongation and desaturation [93,94]. LXRs are activated by oxysterols and cholesterol intermediates and required for basal expression of insulin-mediated *Srebp-1c* transcription and lipogenesis through two LXRs-responsive elements (LXREs) identified on *Srebp-1c* promoter [93,95]. However, *Srebp-1c^−/−^* mice with T0901317 treatment, a LXRs agonist, still express a subset of lipogenic genes and a modest increase in fatty acid synthesis that indicate another mechanism mediated by LXRs can partially activate hepatic lipogenesis [82,94]. The upregulation of lipogenic genes under LXRs agonist administration in *Srebp-1c* null mice are identified to be directly regulated by ChREBP which RNA level is increased in mice receiving the LXR ligand [96]. Expression of ChREBP genes is regulated by several transcription factors and its different isoforms (ChREBPβ itself is induced by ChREBPα) [89]. LXRs is another critical regulator which binds to two LXREs located around 2.4-kbp upstream of the *Chrebp* promoter and activates expression of the *Chrebp* [94]. The influence of hepatic LXRs on expression of *Srebp-1c* and *Chrebp*, in turn, play a key role in many glycolytic and lipogenic genes, including glucokinase (GK), ACCs, FAS, and stearoyl CoA desaturase 1 (SCD1) [97,98,99]. Many small molecule agonists of LXRs have been reported to be effective for treatment of mouse models in metabolic diseases, i.e., diabetes and cholesterol disorders. Treatment with LXR agonists, such as T0901317 and GW3965, reduces the cholesterol level of plasma and the liver and inhibits the development of atherosclerosis in mouse disease models through inducing the expression of target genes of both LXRα and LXRβ and regulating *Srebp-1c* and *Chrebp* in liver and adipose tissue [100,101]. However, both T0901317 and GW3965 have been demonstrated to elevate triglycerides in plasma and liver. Therefore, new potent and effective LXR agonists without the side effects have been developed for clinical usage including a synthetic oxysterol *N*,*N*-dimethyl-3β- hydroxycholenamide (DMHCA), CS-8080, BMS-779788, and LXR-623 [92]. LXRs are now considered fundamental to regulate the systemic homeostasis of lipids, such as sterols, fatty acids, and phospholipids (Figure 1). Elucidating the LXR-dependent biological signals may be a potential therapeutic way for treating metabolic disorders.

## 6. Circadian Clock and NAFLD

In order to adjust the physic state to the light/dark cycle generated by the rotation of the earth, most organisms have developed an endogenous time-keeping system, known as the circadian clock, which generates self-sustained daily fluctuations in behavior and physiological processes. These autonomous clocks are reset every day by sun light, but it can self-sustain circadian rhythm without light stimulation for a long-time. Circadian regulation of cell cycle, hormone secretion, nutrients absorption, and metabolic fluxes coupled to the behavioral timing of sleep/wake (feeding/ fasting) cycles are important to preserve normal function of the liver [102]. Glucose and lipid metabolism in the liver are two critical factors to be reset by feeding behavior which maintains the local integration of systemic and nutritional signals to switch fuel production and utilization for survival. Noteworthy, the light/dark cycle play a role to entrain the central clock, whereas, the feeding/ fasting circuit can reset the peripheral clocks independently of the central clock, which allows the endogenous clock to detect local variations of energy and governs circadian oscillations in metabolism accordingly [103]. Epidemiologic studies show that circadian misalignment increases the risk of developing metabolic diseases such as obesity, type 2 diabetes, and NAFLD in night-shift workers (characterized as chronic circadian misalignment), due to irregular food intake patterns, extended light exposure at night, and reduced sleep duration [104,105,106]. The accumulated evidence in mouse models with circadian gene deficiency also suggest that the loss of circadian genes severely impairs the biological and metabolic function of hepatocytes in the liver [107,108,109]. Moreover, harmful effects on postprandial glucose and insulin levels were observed in controlled circadian misalignment models, which indicated decreased insulin sensitivity after disruption of the circadian clock [110]. A transcriptional–translational feedback loop regulated by the core clock genes was demonstrated as the key regulators of circadian rhythmicity of glycolytic and lipogenic metabolism in liver. The bHLH-Periodic Acid Schiff transcriptional activators CLOCK and BMAL1 induce expression of negative feedback repressors Cryptochrome (*Cry1* and *Cry2*) and Period (*Per1*, *Per2*, and *Per3*) to generate around 24-hr oscillations and affect up to 40% of the genomic transcripts [111]. Disruption of circadian clock can lead to insulin resistance, obesity, and promoting NAFLD-induced hepato-carcinogenesis in mice [112,113,114]. For regulation of glucose homeostasis, loss of Bmal1 function in the liver caused excessive fluctuations in glycemia levels during the post-absorptive phase [115]. Insulin also promotes Akt-mediated Ser42-phosphorylation of Bmal1 to induce its dissociation from DNA, which impairs Bmal1 transcriptional activity [116]. In addition, Cry1 regulates hepatic gluconeogenesis through binding to G protein-coupled receptors and attenuates the transactivation of gluconeogenic genes [117]. In contrast, overexpression of Cry1 in the liver reduces glycemia levels and increases insulin sensitivity in diabetic mice. Moreover, induction of *Per2* by glucocorticoids was demonstrated to affect glucose metabolism in mice with hyperglycemic conditions which are protected from glucose intolerance as lose of glucocorticoid response element in the *Per2* promoter [118]. Moreover, the circadian clock also regulates hepatic lipid metabolism, such as lipoprotein synthesis, lipid uptake and conversion, as well as de novo synthesis and oxidation of fatty acids, conversely, lipids play as a potential regulators for circadian rhythmicity [119]. Lipogenic regulatory factors including peroxisome proliferator-activated receptor (PPAR), LXRs, *Srebp-1c,* PGC1, NR1D1, and ROR display rhythmic expression in liver, which is altered in core clock genes deficient mice [120,121,122,123,124]. Also, NR1D1-mediated circadian signaling via insulin induced gene 2 (INSIG2)–SREBP and LXR has been demonstrated to promotes rhythmic expression of the rate-limiting enzyme cholesterol 7a-hydroxylase (CYP7A1) for cholesterol and lipid metabolism [120]. Therefore, understanding the role of the circadian clock in regulating glycolytic and lipogenic metabolism might shed light on preventing or treating non-alcoholic fatty liver disease (Figure 1). 

## 7. Therapies and Management of NAFLD

Insulin resistance related to metabolic syndrome is the critical pathological process of NAFLD, which is highly associated with genetic variations, lifestyle inactivation, and abnormal food intake. The main treatments for managing type 2 diabetes include lifestyle intervention, pharmacological intervention, and in some surgical cases. Although drugs can relieve the symptoms induced by insulin resistance, and help to fend off complications, the progression of disease cannot be ceased. Lifestyle interventions composing of diet, exercise, and weight loss have been demonstrated to be the key to improving patients with NAFLD. Recently, a case report showed that planned intermittent fasting may help to reverse type 2 diabetes and eliminate the need for insulin treatment [125]. Therapeutic fasting is a kind of caloric restriction that is defined as the voluntary abstinence from all calorie-containing food and drinks during a specified period to promote loss of body weight. In a meta-analysis of randomized trial, people who were able to lose at least 5% of body weight showed improvement in hepatic steatosis, and non-alcoholic steatohepatitis was improved with a loss of 7% body weight [126]. Moreover, a one-year prospective trial with paired liver biopsies in 261 patients demonstrated that the greater the degree of weight loss, the more significant the improvement in the features of NAFLD, including portal inflammation and fibrosis [127]. Therefore, caloric restriction (~840 calories/day) and weight loss (~7%) are important factors for the relief of insulin resistance related to metabolic syndrome and NAFLD.

Unfortunately, only half of patients were able to accomplish a 7% weight loss. Lifestyle modification is not so easy to achieve and to maintain for most patients. Hence, pharmacological medication targeting various aspects of the fat accumulation and injury pathways are always accompanied with lifestyle interventions. Targeting the pathways involved in insulin resistance has been demonstrated as a critical therapeutic regimen for NAFLD progression. The therapies of insulin resistance can be classified based on their intended targets, including bile acid-based insulin sensitization, peroxisome proliferator-activator receptors, FGF21, and metformin. A nuclear bile acid receptor, Farnesoid X receptor (FXR), plays a suppression role in bile acid production by controlling the rate-limiting enzyme cholesterol 7 alpha-hydroxylase (CYP7A1). Importantly, the newest study showed that changes in the bile acid composition were detected in patients with NASH [128]. Obeticholic acid (OCA), a selective FXR agonist, is the first synthetic bile acid for the treatment of NASH that showed the potential anti-inflammatory and anti-fibrotic effects in the liver [129]. Moreover, FXR-mediated release of FGF19 in the intestine has also been demonstrated to improve NASH [130]. Therefore, developing small molecules as FXR agonists with fewer adverse effects are currently a key strategy to combat NAFLD progression. Recently, many peroxisome proliferator-activated receptor (PPAR) α/δ agonists have been identified, such as MBX-8025, elafibranor (GFT-505), and saroglitazar. In a diabetes mouse model, MBX-8025 displays a role to abolish lipotoxicity and improve NASH [131]. In addition, elafibranor has been demonstrated to ameliorate insulin sensitivity in the liver, adipose tissue, and peripheral tissue [132]. Furthermore, in a NASH mouse model, saroglitazar was found to lower steatosis and ALT as well as improve liver histology [133]. In NAFLD patients with dyslipidemia, saroglitazar also significantly decreased in ALT level after 24 weeks administration. Other investigational anti-fibrotic agents, such as FGF21 and metformin, display improvement in hepatic steatosis and a decrease in hepatic stiffness by an increase of insulin-sensitization and normalization of serum aminotransferases, respectively. Together, these studies indicate that the small molecules processing a reduction in liver inflammation and fibrosis as well as an improvement in insulin sensitivity and metabolic complications will be the pharmacological medication targets of anti-NAFLD drug discovery.

## 8. Conclusions

Recent research of the origin of NAFLD under molecular events can help to explain why insulin resistance results in formation of NAFLD and the interplay between NAFLD and insulin resistance. The industrialized society is a critical factor to cause dysregular human activities such as overloading work, excessive dietary intake, and sleep deprivation, which leads to perturbations in metabolism and development of the insulin resistance. Strategies targeted at circadian regulators or lifestyle modification might therefore be used and widely considered as a powerful tool for treatment of liver diseases and against socioeconomic diseases.
